# Vertical and Horizontal Transmission of ESBL Plasmid from *Escherichia coli* O104:H4

**DOI:** 10.3390/genes11101207

**Published:** 2020-10-16

**Authors:** Sandra Daniel, Kelly Goldlust, Valentin Quebre, Minjia Shen, Christian Lesterlin, Jean-Yves Bouet, Yoshiharu Yamaichi

**Affiliations:** 1 Institute for Integrative Biology of the Cell (I2BC), Université Paris-Saclay, CEA, CNRS, 91198 Gif-sur-Yvette, France; sandra.daniel@i2bc.paris-saclay.fr (S.D.); minjia.shen@i2bc.paris-saclay.fr (M.S.); 2 Microbiologie Moléculaire et Biochimie Structurale (MMSB), Université Lyon 1, CNRS, Inserm, UMR5086, 69007 Lyon, France; kelly.goldlust@ibcp.fr (K.G.); christian.lesterlin@ibcp.fr (C.L.); 3 Laboratoire de Microbiologie et de Génétique Moléculaires (LMGM), CBI, CNRS, Université de Toulouse, UPS, 31062 Toulouse, France; valentin.quebre@ibcg.biotoul.fr (V.Q.); jean-yves.bouet@univ-tlse3.fr (J.-Y.B.); 4 Graduate School of Structure and Dynamics of Living Systems, Université Paris-Saclay, CEA, CNRS, 91198 Gif-sur-Yvette, France

**Keywords:** horizontal gene transfer, conjugation, plasmid segregation, post-segregational killing (PSK), toxin-antitoxin (TA), Tnseq, Tn-seq, parABS

## Abstract

Multidrug resistance (MDR) often results from the acquisition of mobile genetic elements (MGEs) that encode MDR gene(s), such as conjugative plasmids. The spread of MDR plasmids is founded on their ability of horizontal transference, as well as their faithful inheritance in progeny cells. Here, we investigated the genetic factors involved in the prevalence of the IncI conjugative plasmid pESBL, which was isolated from the *Escherichia coli* O104:H4 outbreak strain in Germany in 2011. Using transposon-insertion sequencing, we identified the pESBL partitioning locus (*par*). Genetic, biochemical and microscopic approaches allowed pESBL to be characterized as a new member of the Type Ib partitioning system. Inactivation of *par* caused mis-segregation of pESBL followed by post-segregational killing (PSK), resulting in a great fitness disadvantage but apparent plasmid stability in the population of viable cells. We constructed a variety of pESBL derivatives with different combinations of mutations in *par*, conjugational transfer (*oriT*) and *pnd* toxin-antitoxin (TA) genes. Only the triple mutant exhibited plasmid-free cells in viable cell populations. Time-lapse tracking of plasmid dynamics in microfluidics indicated that inactivation of *pnd* improved the survival of plasmid-free cells and allowed *oriT*-dependent re-acquisition of the plasmid. Altogether, the three factors—active partitioning, toxin-antitoxin and conjugational transfer—are all involved in the prevalence of pESBL in the *E. coli* population.

## 1. Introduction

Commensal, harmless bacteria can turn into pathogens by acquiring virulence factors. Bacteria can also gain resistance to certain antibacterial agents, together with or independently of virulence factors. Virulence/resistance genes can move from one cell to the other via mobile genetic elements (MGEs) such as transposons, prophages, integrative and conjugative elements (ICEs) and plasmids. Many plasmids and ICEs transfer through cell-to-cell contact by a mechanism referred to as bacterial DNA conjugation. These transferrable genetic elements encode genes required for conjugational gene transfer, including conjugative pili/Type IV secretion machinery for ‘mating pair formation’, as well as DNA processing proteins that process the double-stranded DNA (dsDNA) to generate single-stranded DNA (ssDNA) from the origin of transfer (*oriT*) site [1]. Both the strand that was transferred into the recipient and the strand that remained in the donor subsequently are converted into dsDNA by complementary strand synthesis replication. Virulence and drug-resistance plasmids are often conjugative plasmids, and the capacity of the recipient to turn into a new donor cell contributes to their dissemination. Yet, establishment and maintenance of MGEs in host cells are also critical steps for their prevalence in a cell population. When MGEs are integrated into a host chromosome (e.g., ICE), they are rather stably inherited, as bacteria faithfully segregate their chromosomes to daughter cells. On the contrary, plasmids that are in the cytoplasm replicate and segregate independently to the host chromosome, and so are in danger of being lost at every cell division cycle. This is particularly the case for conjugative plasmids because they are typically large and kept at low-copy-numbers to reduce the metabolic burden for bacterial host cells.

To avoid the dilemma, conjugative plasmids harbor fail-safe mechanisms to ensure their stable presence and inheritance at the population level. The first mechanism is known as active partitioning where subcellular machinery pushes or pulls the plasmid copies towards the opposite halves of the cell so that each daughter cell receives a copy at cell division (see [2,3,4] for review). Based on the motor NTPase component, plasmid partitioning systems have been classified in three general types: Type I (ParABS), Type II (ParMRC) and Type III (TubZRC) utilize Walker-type ATPase ParA, Actin-like ATPase ParM and tubulin-like GTPase TubZ, respectively. ParB, ParM and TubR are DNA-binding proteins that specifically bind to the centromeric sites *parS*, *parC* and *tubC*, respectively, which consist of direct and/or inverted repeats located downstream and/or upstream of their respective *parAB/parRM/tubRZ* operons. Among the three types, Type I is the most prevalent in plasmids and can be divided into Ia and Ib sub-types according to the size of the ParB protein [2,3,4,5]. Furthermore, Type Ia partitioning genes are also found in many bacterial chromosomes. Interestingly, nucleotide sequence of chromosomal *parS* is very much conserved, whereas that of extra-chromosomal *parS* (including secondary chromosomes of multi-chromosomal bacteria) is highly variable [4,6].

The second mechanism to maintain the plasmid in the host cell is the toxin-antitoxin (TA) system, which is also known as post-segregational killing (PSK) or plasmid addiction. Literally, the plasmid encodes the toxin and its neutralizing antidote, and once the occasional event of mis-segregation happens, a plasmid-free daughter cell suffers from the lasting toxin because the cognate antitoxin is relatively unstable and cannot be regenerated. There are diverse modes of action for toxins (disrupt membranes, inhibit protein synthesis, inhibit DNA replication, etc.) and antitoxins (sRNAs or proteins prevent translation of the toxin gene, degrade/neutralize the toxin, etc.) [7,8]. Like an active partitioning locus, TA loci are also found on bacterial chromosomes, often associated with MGEs [9]. Chromosomal TA has been suggested as involved in stress response, bacterial growth arrest and persistence; however, this view has been challenged and debated [10].

In the outbreak of enterohemorrhagic/enteroaggregative *Escherichia coli* in Germany in 2011, the causative strain encoded Shiga-toxin, the key virulence factor for diarrhea, on a prophage integrated into the chromosome. In addition, it commonly harbored two plasmids, pAA and pESBL. pAA mediates aggregative adherence, a feature of enteroaggregative *E. coli*, and pESBL confers resistance to beta-lactam antibiotics [11,12]. While pAA is dispensable and can be lost from the host *E. coli*, pESBL was shown to present utmost stability [13,14,15,16]. Besides, pESBL was shown to transmit among Enterobacteria such as between *E. coli* and from *E. coli* to *Klebsiella*, by conjugation [14], which is consistent with the ‘plasmid host range’ of IncI conjugative plasmids. 

Transposon-insertion sequencing (Tnseq) combines transposon mutagenesis and next generation sequencing (NGS) methodologies to assess essentiality and/or fitness contribution for genes and genetic loci in given growth conditions [17]. Genes essential for replication and maintenance have been identified by Tnseq in an IncA/C plasmid that is responsible for multidrug resistance (MDR) of an uropathogenic *E. coli* strain [18]. For pESBL, genes important for conjugative transfer have been identified by comparing transposon insertion libraries before and after conjugational transfer [14]. However, solely inspecting the initial library (before conjugational transfer) has resulted in identification of the replication locus (*repYZ*) but not the maintenance loci. Since pESBL shows considerably higher efficiency for conjugational transfer [14,19], it is also conceivable that plasmid loss can be compensated for by following conjugational transfer, if cells are not affected by PSK.

In this study we characterized plasmid factors contributing to pESBL stability in the *E. coli* host. We found that pESBL encodes a Type Ib active partitioning system with novel ParB and *parS*. Combination of mutations for *pnd* TA and plasmid mobilization revealed that all three factors are involved in pESBL stability. These results suggest that plasmid loss can be offset by re-entering of plasmid from neighboring cells. Indeed, we were able to capture mis-segregation as well as conjugational transfer events under the microscope.

## 2. Materials and Methods

### 2.1. Standard Microbiology Procedures

Bacterial cells were grown in lysogeny broth (LB) media and antibiotics were used at the following concentrations when appropriate and not otherwise noted: ampicillin (Amp), 100 µg/mL; chloramphenicol (Cm), 25 µg/mL; kanamycin (Km), 50 µg/mL; and streptomycin (Sm), 100 µg/mL. A list of strains, plasmids and oligo DNAs used in this study are shown in Supplementary Tables S1–S3, respectively. The mCherry-ParB_pMT1_ expression strain was constructed as follows: *mCherry-ParB_pMT1_* flanking the *kan* gene was amplified from pEYY373 and introduced to the *galK* locus of the *E. coli* chromosome using the lambda Red recombination system [20]. The resulting *∆galK::mcherry-parB_pMT1__kan* was moved to fresh MC1061 background by P1 transduction, resulting in bEYY2125. For a mini-F stability test, potential pESBL *par* locus (*par_pESBL_*) was amplified with oYo1032 and oYo1033 followed by cloning into the *Bam*HI site of pXX705 by Gibson assembly (New England Biolabs, Ipswich, QLD, USA). For electrophoretic mobility shift assay (EMSA) of the ParB/*parS* interactions, the *parB* (*hp7*) gene was cloned into pET28b by Gibson assembly. The resulting pEYY395 was introduced to *E. coli* BL21(DE3). The allelic exchange gene replacement method was used to introduce insertions/deletions in pESBL [14,19]. In brief, ∆*oriT*, ∆*par* and ∆*pnd* correspond to deletions of the 121 bp *oriT* locus, *parB*-O3K_28522 ORFs and the *pndB-pndA* (O3K_25987) region, respectively. The construction of plasmids is detailed in Supplementary Table S2. Conjugation was used to move pESBL mutants to different *E. coli* backgrounds, except for ∆*oriT* mutants of pESBL, for which P1 transduction was used to move chromosomal mutation, or the *oriT* deletion, which was introduced last. Transfer efficiency and plasmid stability tests were carried out as described in [19] and [21], respectively. In brief, the transfer efficiency assay was carried out with 100 and 10 μL of overnight cultures of recipient and donor cells, respectively. Cells were washed and mixed on the top of a 0.45-μm HAWP filter (EMD Milipore, Burlington, VT, USA) on LB agar plate. After incubation at 37 °C for 2 h, cells were recovered and plated on LB agar plates containing relevant antibiotics with appropriate dilutions. For the plasmid stability assay, *E. coli* cells harboring the test plasmid were subjected to growth without Amp selection. At various time points, the culture was back-diluted to keep cells in the exponential growth phase as well as plated to isolate colonies. At each time point, 100 colonies were tested for the retention of plasmids by plating to an LB agar plate containing 25 µg/mL Amp. Cell growth rates were measured with a microplate reader (Tecan Infinite M200 Pro, Tecan group, Männedorf, Switzerland) [19]. For the fitness assay, overnight cultures of *lacZ*^+^ and *lacZ*^−^ strains were mixed in 1:1 a ratio and grown in LB broth. At various time points, aliquots of cells were spread on LB plates containing 40 µg/mL of X-gal to count Lac^+^ and Lac^−^ cells. Cultures were also back-diluted to keep cells in the exponential growth phase. Competition index (CI) was measured as the ratio of Lac^−^/Lac^+^ (or Lac^+^/Lac^−^) at each time point divided by that of time 0.

### 2.2. Transposon-Insertion Sequencing

Transposon (Tn) insertion mutant libraries (i.e., ‘input’ library) were prepared essentially as described in [14], except we used the *MmeI+* derivative of *Himar1* Tn with pEE18. From the input library, ~230,000 cells were subjected to the plasmid loss experiment where cells were grown in LB broth with Sm and Km but without Amp. The OD of the culture was periodically measured to calculate the growth rate, and accordingly back-diluted to keep cultures in the exponential growth phase. After 12 h (~25 generations) of incubation, cells were plated on LB agar containing Sm, Km and Amp and ~890,000 colonies were scraped to collect as the ‘output’ library. NGS library preparation, including gDNA preparation, MmeI digestion, adapter ligation and PCR amplification, was carried out as described in [22]. Three independent libraries were prepared with distinct ‘barcodes’ and subjected to independent Illumina sequencing twice, which was carried out at the I2BC NGS facility (Gif-sur-Yvette, France). Single-end, 75-bp-reads were proceeded for the data analysis.

### 2.3. Transposon-Insertion Sequencing Data Analysis

After removing P5 adapter and Tn sequences by CutAdapt [23], the resulting ~16 nt reads were mapped to the *E. coli* MG1655 (chromosome) and pESBL-EA11 (pESBL) sequences using Bowtie [24]. If a read mapped multiple times on the reference genome (chromosome + pESBL) (e.g., at rRNA and IS), we randomly distribute it to one of the sites. Mapped reads at every TA site were counted and visualized with the Artemis genome browser [25]. Previous annotation of pESBL was used [14]. Summary of Tnseq data analysis, including barcode and adaptor information, is shown in Supplementary Table S4.

### 2.4. Electrophoretic Mobility Shift Assay

Cell extracts from strains BL21 (DE3) carrying the pEYY395 or pET28b vector were produced as previously described (Supplementary Figure S1) [26]. In brief, overnight cultures in LB containing Km were diluted 100-fold in 50 mL of fresh LB with Km and incubated at 37 °C. At OD_600nm_ ~ 0.5, protein expression was induced by adding 0.1 mM Isopropyl β-D-1-thiogalactopyranoside (IPTG) and cultures were further incubated for 4 h at 37 °C. Cells were chilled on ice, harvested by centrifugation, washed in cold TNE buffer (50 mM Tris–HCl pH 7.5, 50 mM NaCl, 1 mM EDTA), resuspended to an OD_600nm_ ~ 200 in TNE supplemented to 200 mM NaCl and containing lysozyme (500 µg/mL) and left on ice for 30 min. Viscosity was then reduced by sonication before the cell-free extracts were subjected to centrifugation at 22,000× *g* for 20 min at 4 °C. Aliquots of the supernatants were then frozen rapidly in liquid nitrogen and kept at –80 °C until use. Non-specific (ns), seven direct repeat (7-DR) and eight repeat (8-BS) dsDNA probes were prepared by annealing complementary PAGE-purified oligo nucleotides ns-F + ns-R, 7DR-F + 7DR-R and 8BS-F + 8BS-R, respectively, where forward (F) strands are labeled with Cy3 at the 5’ end (Supplementary Table S4). Binding reaction (10 µL) consists of 4 pmol of Cy3-labeled DNA probes in a buffer containing 50 mM Tris-HCl (pH 7.5), 100 mM NaCl, 250 µM DTT, 15% glycerol, 1 mM EDTA, 100 µg/mL of salmon sperm DNA and cell extract. The range of ParB level was obtained via serial dilution of the ParB-induced cell extract into the cell extract without ParB (Supplementary Figure S1), with the range “100” and “1” corresponding to no dilution and 100-fold dilution, respectively. After 15 min incubation at 24 °C, the reactions were then electrophoresed on 5% polyacrylamide gels in TGE buffer (25 mM Tris base, 190 mM glycine, 1 mM EDTA) supplemented with 8% glycerol. Electrophoresis was performed at 170 V for 170 min at 4 °C. The gels were then revealed using a Typhoon Trio (GE-Healthcare, Chicago, IL, USA) with a 532 nm wavelength emission laser and 580 BP 30 filter.

### 2.5. Microscopy

For microscopy analyses, cells were grown in M9-Glucose media supplemented with casamino acids. For Snapshot analysis, 1 µL of an exponentially growing cell culture was spotted on the agarose pad (1% w/v) and phase contrast and GFP signals were acquired using a DM6000-B (Leica Microsystems, Wetzlar, Germany) microscope. For the microfluidics, the culture was prepared with M9-Glucose media supplemented with 0.4% casamino acids and Amp at 37 °C overnight. Cells were back-diluted to a starting OD_600 nm_ of 0.05 in fresh media without Amp selection. After 2 h of growth to allow the potential emergence of plasmid-free cells, the culture was loaded into a B04A microfluidic chamber (ONIX, CellASIC, EMD Milipore) preheated at 37 °C. A continuous flow of fresh media without Amp was injected at 2 psi into the microfluidic chamber throughout the experiment (2 h) at 37 °C. Wide-field microscopy imaging was carried out on an Eclipse Ti-E microscope (Nikon, Tokyo, Japan), equipped with × 100/1.45 oil Plan Apo Lambda phase objective, FLash4 V2 CMOS camera (Hamamatsu Photonics, Hamamatsu, Japan), and using NIS software (Nikon) for image acquisition. Phase contrast and mCherry images were acquired every 15 min with 50 ms and 100 ms exposure, respectively Resulting images were analyzed with Fiji/ImageJ software [27].

## 3. Results

### 3.1. Transposon-Insertion Sequencing

**Figure 1:**
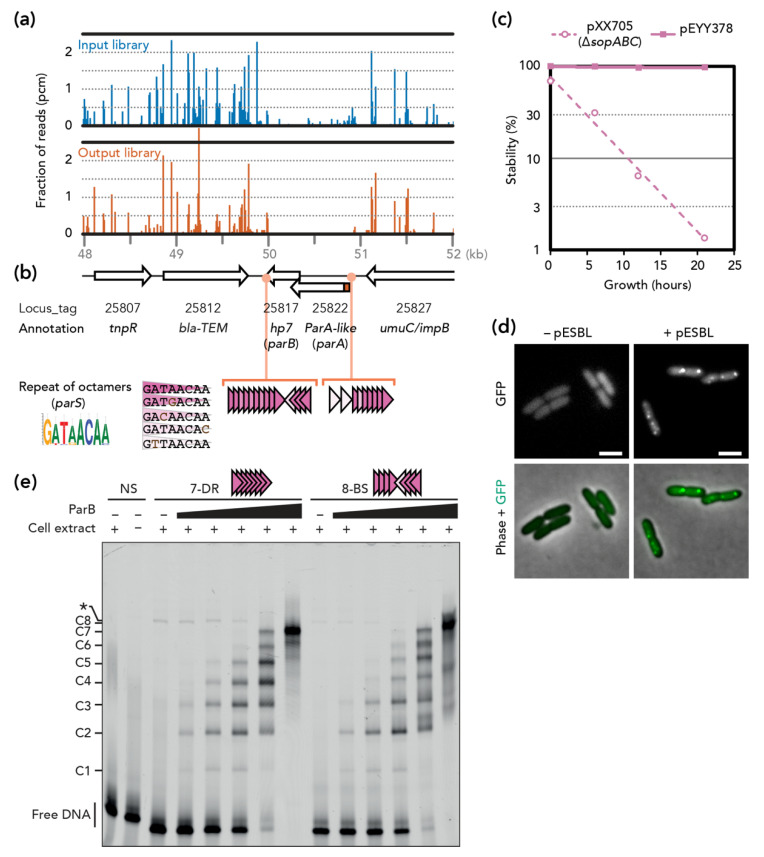
pESBL partitioning locus. (**a**) Tnseq reads mapped on the partitioning locus and its flanking region. Fraction of reads corresponds to the number of reads obtained for each TA dinucleotide divided by the total number of reads mapped to pESBL. (**b**) Schematic map of the region. N-terminal extension of pESBL ParA (see text) is shown in red. Consensus sequence for the octamer nucleotide is shown in the sequence logo format [28]. Triangles indicate octamer repeat with or without a mismatch to the consensus sequence. (**c**) Stability of mini-F plasmids. (**d**) Representative microscopy images of GFP-Hp7 (ParB) in MC1061 with (+) or without (–) pESBL. Bars = 2 µm. (**e**) EMSA. dsDNA probes were incubated (+) or not (–) with cell extract containing increasing amounts of ParB (black triangle with a range of 1, 3, 10, 30, 100). The positions of various protein–DNA complexes (C1-C8) and free DNA fragments are indicated on the left. A non-specific complex, indicated by an asterisk (*), is observed in ParB-free extract with probes 7-DR and 8-BS but not with non-specific DNA (NS) (Figure S1b).

Tnseq experiments comparing two transposon insertion libraries were designed to identify pESBL maintenance loci, where inactivation by transposon insertion would result in plasmid loss. Previous Tnseq experiments to identify transfer genes indicated that certain transposon insertion mutants exhibited extremely enhanced transfer efficiency [14]. To avoid any chance of re-transfer of pESBL, we carried out Tnseq in a ∆*oriT* background that completely abolished transfer ability (Supplementary Figure S2). The Tn insertion mutant library of *E. coli* cells harboring pESBL ∆*oriT* was prepared (‘input’ library) and cells were grown without Amp selection for ~25 generations, allowing unstable mutants to be lost from the population (‘output’ library). When the two Tnseq results were compared, a ~1 kb region of pESBL was found to have very few Tn mutants in the output library compared to the input library (Figure 1a).

In this region, two ORFs, O3K_25822 and O3K_25817, were deduced. O3K_25822 was originally annotated as ‘Type II plasmid partitioning protein’, but was later revised to a ParA-like protein [14]. Indeed, the predicted amino acid sequence did not contain actin-like but a Walker-type ATPase motif B. ATPase function requires both Walker A and B motifs, and the former was lacking in the O3K_25822 ORF. However, investigation of the nucleotide sequence in this region suggested that translation of O3K_25822 could start at an alternative start codon 93 bp upstream. Remarkably, the resulting 31 amino acid-long extension in the N-terminus included a Walker A motif. On the other hand, O3K_25817 was annotated as a hypothetical protein (Hp7) and neither shared any homology to known ParB proteins nor harbored a predicted DNA binding motif. This is not unusual, since ParB of Type Ib are poorly conserved [4]. Lastly, we found repetitive octamer nucleotides both upstream and downstream of the ORFs that were reminiscent of *parS* sites (Figure 1b).

### 3.2. pESBL Par Locus

Although Tnseq results, organization of genes, and homology of ParA collectively suggest that this region encodes a potential Type Ib plasmid partitioning system, O3K_25817 and the octamer repeats do not have sequence homology to known ParB and *parS*, respectively. To test whether this region is a bona fide partition locus and functions for plasmid stability, we first tested plasmid stability in a heterologous system. Low-copy-number mini-F plasmid has been used widely for this purpose. Mini-F plasmid containing its own partitioning genes (*sopABC*) is extremely stable in *E. coli* cells, while lack of *sopABC* results in a rapid loss of the plasmid in the population of *E. coli* [29]. This instability can be restored by a non-native plasmid/chromosome partitioning system (ParA and ParB can be provided in trans but *parS* has to be cloned in *cis*) [21,30,31,32]. As shown in Figure 1c, cloning of the potential pESBL partitioning locus in the unstable pXX705 conferred high stability to the resulting pEYY378.

Next, we used an in vivo microscopic approach for further validation of potential ParB. It is known that fluorescent protein fusion of ParB displays discrete foci when a cognate *parS* site is present in the cells [21,33,34]. Accordingly, we constructed an expression plasmid encoding *gfp-hp7*, and the fusion protein was ectopically expressed in *E. coli* cells that both contained and did not contain pESBL. GFP foci were apparent in *E. coli* harboring pESBL, but the signal was diffuse in cells without pESBL (Figure 1d). 

Lastly, ParB binding to the potential *parS* sites (i.e., octamer repeats) was investigated in vitro. The EMSA experiments clearly showed that ParB specifically binds to repeats of the octamer sequence, either in direct repeats (7-DR) or on two arrays of four direct repeats in opposite orientations (8-BS) (Figure 1e and Supplementary Figure S1). As the amount of ParB increases, the number of bound complexes increases gradually up to the number of repeats present in the probes, (i.e., seven and eight discrete complexes for the probes with seven and eight repeats, respectively). These results suggest that one ParB could bind to each octamer repeat.

Altogether, these results indicate that this region encodes pESBL partitioning genes, and hereafter we called O3K_25822 (with N-terminal extension), O3K_25817, and octamer repeats ParA, ParB, and *parS* of the pESBL, respectively.

### 3.3. Stability and Fitness ∆par Mutants

**Figure 2:**
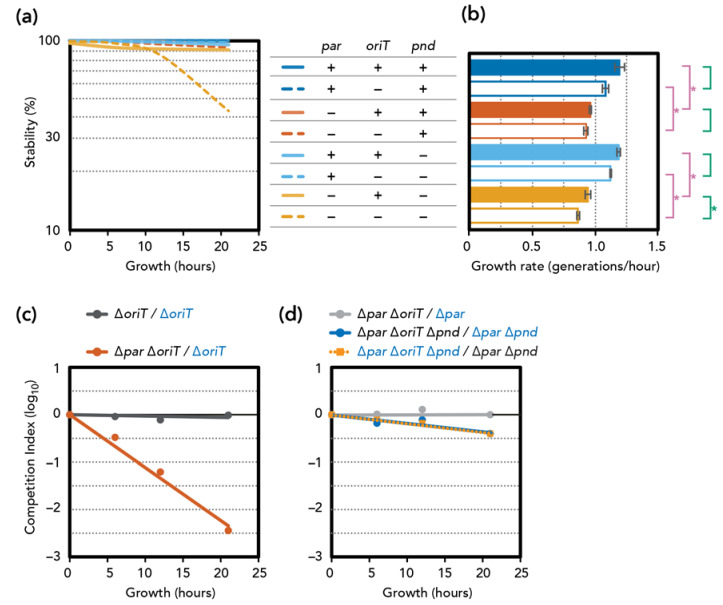
Phenotypes of pESBL mutants. (**a**) Stability of various pESBL mutants: presence and absence of genes are indicated by + and−, respectively. Averages of three independent experiments are shown. (**b**) Growth rate of pESBL mutants measured by plate reader. Average and standard deviations of three independent measurements are shown. For comparison between *parABS* +/− (in magenta) and *oriT* +/− (in green) counterparts, * denotes *p* < 0.01 in a two-tailed Student’s *t*-test. (**c**,**d**) Competition index (CI) between two co-cultured mutants. *lacZ^+^* derivatives are indicated in blue. The ratio of colony forming units was normalized to that of time 0, and an average of three independent experiments is shown.

We constructed deletion mutants of the pESBL partitioning locus. Unexpectedly, plasmid stability assays indicated that ∆*par* mutants remained highly stable. (Figure 2a). However, we noticed that deletion of the *par* locus is also associated with a significant delay in growth rate compared to the *par*^+^ counterparts (Figure 2b). This growth defect was more evident in competition assays where we used the LacZ^+^ phenotype to distinguish two plasmids. We first ascertained that inserting a *lacZ* gene at an intergenic region of pESBL did not significantly alter transfer efficiency (Supplementary Figure S2) and fitness (Figure 2c, *∆oriT/∆oriT lacZ^+^*). Competition assay showed that cells harboring the ∆*par* ∆*oriT* mutant were rapidly eliminated from the co-culture with cells harboring ∆*oriT* pESBL (Figure 2c). These results are consistent with the idea that *par* deletion caused mis-segregation of the pESBL, and only the daughter cells that received the plasmid could survive, while daughter cells that lost the plasmid were eliminated by PSK.

Many IncI conjugative plasmids encode the *pnd* TA system, of which *pndB* antisense RNA neutralizes PndA membrane-damaging toxin [35,36,37,38]. Indeed, pESBL also encodes *pnd* genes in the conserved transfer locus, and we tested the effect of *pnd* deletion in both wild-type and ∆*par* backgrounds. Unlike *par* deletion, the ∆*pnd* mutation alone did not affect plasmid stability or the growth rate of *E. coli* cells harboring the mutant plasmid. Analysis of the ∆*par* ∆*pnd* double mutant showed a slight decrease in plasmid stability, which can still be considered stable as ~90% of the cells retained the plasmid after 21 h of growth without selection (Figure 2a). Furthermore, ∆*pnd* did not suppress the viability defect induced by the ∆*par* mutation (Figure 2b).

### 3.4. Involvement of Plasmid Transfer

**Figure 3:**
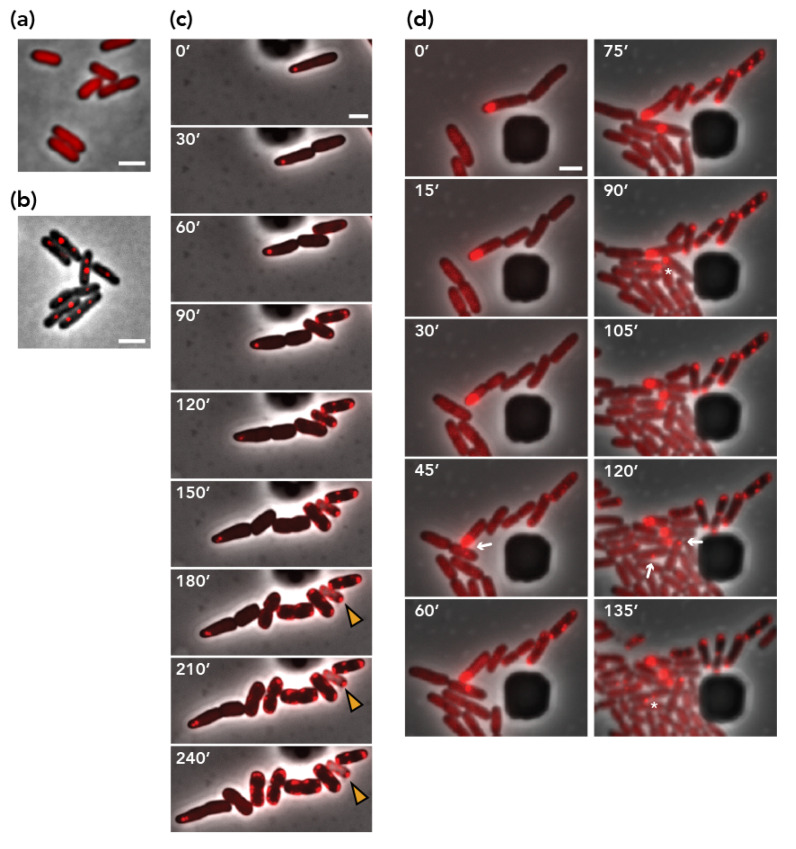
pESBL plasmid dynamics. (**a**,**b**) Representative image of *E. coli* cells expressing mCherry-ParB_pMT1_, in the absence (**a**) or presence (**b**) of *parS*_pMT1_^+^ pESBL. (**c**) Representative time-lapse images showing mis-segregation of ∆*par* ∆*pnd parS*_pMT1_^+^ pESBL. Arrowheads indicate dead cells that lost membrane integrity. (**d**) Representative time-lapse images showing the re-transfer of ∆*par* ∆*pnd parS*_pMT1_^+^ pESBL. Conjugational and vertical transfer events are indicated with arrows and asterisks, respectively. Bars = 2 µm.

Next, we performed a microscopic visualization of pESBL molecules to follow plasmid dynamics in *E. coli* cells. To this end, we used a *parS*/ParB DNA-labeling system with a *parS*/ParB pair from the pMT1 plasmid, which does not cross-react with distinct *parABS* [33,39]. bEYY2125, which expresses *mCherry-parB_pMT1_* from a *galK* promoter, exhibited diffuse red signals over the nucleoid (Figure 3a). When *parS_pMT1_* was introduced in an intergenic region of pESBL and moved into bEYY2125, mCherry-ParB_pMT1_ made discrete foci inside the cell (Figure 3b). We also constructed a ∆*par* ∆*pnd* double mutant of *parS_pMT1_*^+^ pESBL and transferred it into bEYY2125. Resulting bEYY2166 often harbored a single mCherry focus near the cell pole, suggesting the mispositioning of the mutant pESBL copies (Figure 3c, 0’). Time-lapse imaging suggested that bEYY2166 cells often divide into two daughter cells, one of which retained the mCherry focus corresponding to the plasmid while the other did not. Remarkably, plasmid-free daughter cells did not divide further, but exhibited a characteristic phenotype with an accumulation of mCherry signals at the cell periphery. Phase contrast images also suggested some of them lost cell integrity (Figure 3c). Further time-lapse microscopy showed that plasmid-free daughter cells that exhibited aberrant mCherry signal accumulation were also sensitivity to Amp (Supplementary Figure S3). These observations clearly confirm our previous interpretation that pESBL plasmid loss induced by *par* deletion is associated with PSK of plasmid-free daughter cells. It also showed that PSK is not strictly attributable to *pnd* TA, suggesting that pESBL might conceal another TA system.

Lastly, we introduced the transfer-deficient mutation ∆*oriT.* The ∆*oriT* mutation had no effect on plasmid stability, except the ∆*par* ∆*oriT* ∆*pnd* triple mutant, which showed clear plasmid instability (Figure 2a). In addition, the effect of ∆*oriT* on growth rate was also evident only when active partitioning and *pnd* TA were both defective (Figure 2b). This result was further supported by co-culture competition assays. While introducing ∆*oriT* in the ∆*par* (TA+) background did not have any impact on fitness, the ∆*par* ∆*oriT* ∆*pnd* triple mutant consistently showed a fitness disadvantage compared to the ∆*par* ∆*pnd* double mutant (Figure 2d). Note however that the plasmid-loss rate and disadvantage of fitness of the triple mutant remained somewhat modest (discussed below). Collectively, these results suggest a scenario that when plasmid mis-segregation occurs due to ∆*par*, the TA will eliminate cells that have lost the plasmid. *pnd* deletion permitted some of them to survive, and they can be the recipient of the conjugative (re-)transfer of pESBL. Indeed, the ∆*par* ∆*pnd* double mutant (thus transfer-proficient) plasmid showed fully restored plasmid stability (Figure 2a).

### 3.5. Visualization of Plasmid Re-Transfer after Mis-Segregation

Using live-cell microscopy, we were able to capture plasmid re-acquisition events in the strain harboring the ∆*par* ∆*pnd* mutant of pESBL. Populations of bEYY2166 cells consisted of plasmid-harboring cells with a discrete mCherry-ParB_pMT1_ focus and viable plasmid-free cells characterized by a diffuse mCherry signal. When a plasmid-harboring cell approached a plasmid-free cell, emergence of an mCherry focus in the plasmid-free cell was observed (Figure 3d 30’ ~ 45’), strongly suggesting conjugational transfer of the pESBL molecule. Furthermore, we were able to follow successful partitioning, mis-segregation, and conjugational transfer events from the recipient and its progeny cells (Figure 3d). In contrast, transfer events were never observed with the ∆*par* ∆*oriT* ∆*pnd* triple mutant plasmid.

## 4. Discussion

Prevalence of virulence and MDR plasmids are one of the major threats to public health. Virulence plasmids can be fully or partially responsible for the pathogenicity of the host pathogen (e.g., pINV of enteroinvasive *E. coli* and pSLT of *Salmonella enterica* serovar typhimurium, respectively), while MDR plasmids can confer on host cells a great fitness advantage when they are challenged by corresponding antibiotics. Note that composite plasmids carrying both virulence and MDR genes are also emerging [40,41]. In virulence plasmids, the presence of active partitioning, PSK and multimer resolution mechanisms ensures their prevalence [42]. Here, we studied maintenance strategies of an MDR conjugative plasmid, pESBL, which is associated with a devastating outbreak of pathogenic *E. coli.* pESBL encodes two broad spectrum beta-lactamases, enabling host cells to be resistant against these antibiotics. Even without antibiotic selection, pESBL was shown to be stably maintained in the host cell in both in vitro (test tube) and in vivo (animal infection model) cultivations [13,14,15,16]. Furthermore, pESBL is suggested to help persistence of the host *E. coli* in ruminant intestine, thus supporting this environmental reservoir of the pathogen [16].

Tnseq and further characterization strongly suggested that two ORFs, O3K_25822 and O3K_25817, along with octamer repeats constitute the active *par* locus of pESBL. It is classified as a Type Ib partitioning system, as it comprises a ParA Walker-type ATPase, a small (100 amino acids) sequence-specific DNA binding protein ParB and two arrays of ParB-binding *parS* sites located both upstream and downstream of the potential operon (Figure 1). Other type Ib Par systems, such as those of pB171 and pSM19035, also harbor dual arrays of direct repeat sequences present both upstream and downstream of their respective *par* operons [43,44]. In addition to putative centromere activity, the upstream *parS* array from pB171 was shown to be involved in transcriptional regulation of the *par* operon [45]. Whether the upstream *parS* array of pESBL is involved in partition and/or gene expression remains to be determined. Type I ParA proteins share common Walker motifs including an N-terminal Walker A motif [30]. In retrospect, proper annotation of ParA_pESBL_ would have facilitated the identification of the pESBL *par* locus. It has been acknowledged that misannotations of plasmid genes/ORFs are a big concern and they should be corrected [46,47,48]. In contrast to ParA, Type I ParB proteins are more divergent. Type Ia ParB proteins are closely related to each other [4] and more recently have been characterized as CTP hydrolase [49,50], whereas Type Ib ParBs do not share an amino acid sequence homology with other members [4], nor do they appear to have CTPase activity. With regard to *parS,* nucleotide sequence of chromosomal *parS* is very much conserved (16 bp for two inverted repeats of octamer) [6]. In contrast, plasmid *parS* is much more divergent in both the nucleotide sequence of each unit and the number and direction of units at each *parS* locus [4]. In pESBL, the octamer sequence constituted direct and inverted repeats upstream and downstream of *parAB* genes. EMSA showed that ParB_pESBL_ can bind to both direct and inverted repeats of the octamer, and affinity to direct and inverted repeats seems comparable (Figure 1e). The consensus sequence of the octamer, GATAACAA, can be found in the *E. coli* chromosome about 250 times. However, GFP-ParB_pESBL_ did not make foci in *E. coli* cells without pESBL (Figure 1d). In EMSA experiments, intensity of protein-DNA complex C1 is fainter than C2, even with lower ParB_pESBL_ concentrations (Figure 1e). It is suggested that ParB_pESBL_ binding to a single octamer is not strong and that cooperative binding to multiple octamer sequences could be important for steady ParB-*parS*_pESBL_ binding. Indeed, on the *E. coli* chromosome, there are no pairs of the octamer forming direct or inverted repeats (minimum 50 bp apart and median 11 kb). Altogether, it is likely that direct/inverted repeats are critical for ParB_pESBL_ binding, similar to other ParABS systems.

During time-lapse microscopy imaging of ∆*par* ∆*pnd* pESBL, characteristic accumulation of mCherry signals at cell peripheries always followed mis-segregation of pESBL (Figure 3c,d). It is possible that pESBL encodes additional TA genes other than *pnd*. Plasmid stability assays also support this hypothesis. ‘Unstable’ plasmids are known to be removed very quickly from the population. For example, in pXX705 mini-F plasmid that lacks active partitioning (*sopABC*), TA (*ccd*) and transfer loci, the fraction of plasmid-harboring cells fell as low as a few percent after 21 h incubation without selection. In contrast, the ∆*par* ∆*pnd* ∆*oriT* triple mutant of pESBL that remained in ~40% of the cells after 21 h (compare Figure 1c and Figure 2a). Unfortunately, annotation of pESBL did not suggest any candidate genes for additional TA. Since Tn insertion in an antitoxin gene could liberate toxin followed by impeding the growth of the mutant, it is possible that a gene classified as ‘essential’ in Tnseq [14] can be an antitoxin gene. 

Tracking subcellular localization of plasmid provides vital information for plasmid dynamics. Here we used ParB/*parS* from the pMT1 labeling method, in which one copy of the 86-bp *parS_pMT1_* site is enough to visualize a plasmid/chromosomal locus as a discrete focus with a fluorescent protein fusion of ParB_pMT1_. Just as the ParB/*parS*_pMT1_ pair does not cause incompatibility, the insertion of *parS_pMT1_* to an intergenic region of pESBL, and binding of mCherry-ParB_pMT1_ to *parS*_pMT1_^+^ pESBL, had at most modest effects on conjugational transfer (Supplementary Figure S2) [33,51]. With this method, we often see that ∆*par* pESBL localized near the cell pole (Figure 3), which is consistent with other ∆*par* mutant plasmids that are known to be excluded from the nucleoid in the cell [29,52,53,54,55]. Interestingly, an IncW plasmid R388 harbors novel (other than Type I, II and III) partitioning genes *stbAB,* and the *∆stbA* mutant R388 localized near the cell poles, resulting in an unstable but 50-fold increase in transfer efficiency [52]. In contrast, we did not see a significant impact of ∆*par* on plasmid transfer efficiency of pESBL (Supplementary Figure S2). Plasmid DNA must be recruited to the cell membrane during conjugation, while active partitioning sustains plasmid associated with nucleoid in the cell. It is intriguing how conjugative plasmids balance active partitioning and conjugative transfer. 

Consistent with the genetics results, we were not able to observe any conjugation events with ∆*oriT* mutant of pESBL. Furthermore, we observed *oriT^+^* pESBL transfer only to plasmid-free cells (Figure 3d). In conjugative plasmids and ICEs, it is known that they prevent conjugational intake of another MGE of the with same exclusion group, including sibling molecules, by entry (surface) exclusion [56,57,58]. IncI plasmids encode *excA-traY* entry exclusion genes and so does pESBL [14,58]. Accordingly, it is credible that conjugative transfer would not work to increase plasmid copies in plasmid-harboring cells but can only compensate the plasmid loss. Therefore, active partitioning would play a primal role in stability.

A worrisome dissemination of MDR plasmid by conjugative plasmid is, in part, due to its ability to turn exconjugant cells into new donor cells. Indeed, we can point out that conjugational transfer events in Figure 3d (120’) happened from a donor cell that had received plasmid earlier (45’) in the time-lapse experiment. Remarkably, several studies, including vintage genetics, have suggested that cells that have newly acquired plasmid are more potent donors than cells already harboring the plasmid [59,60,61]. It is also interesting to see whether one donor cell is capable of transferring plasmid to more than one recipient cell. Use of highly transmissible plasmids such as pESBL and a microfluidics environment would provide a great opportunity to address these fascinating and important transfer dynamics questions.

## Supplementary Materials

The following are available online at https://www.mdpi.com/2073-4425/11/10/1207/s1, Figure S1: Crude cellular extracts and EMSA in the absence of ParB, Figure S2: Transfer efficiency of pESBL mutants, Figure S3: Representative time-lapse images of the fate of cells that have lost ∆*par* ∆*pnd parS*_pMT1_^+^ pESBL, Table S1: Strains used in this study, Table S2: Plasmids used in this study, Table S3: Oligo DNAs used in this study, Table S4: Summary of Tnseq. (References [62,63,64,65] are cited in the supplementary materials)
