# Supplementary material for: Vertical and Horizontal Transmission of ESBL Plasmid from Escherichia coli O104:H4
Source: Genes (Basel). 2020 Oct 16;11(10):1207. doi: 10.3390/genes11101207 (PMC7602700; doi:10.3390/genes11101207)
Supplement: Supplementary file 1 [file genes-11-01207-s001.pdf]

genes

MDPI

Vertical and horizontal transmission of ESBL plasmid from *Escherichia coli* O104:H4

Supplementary Materials:

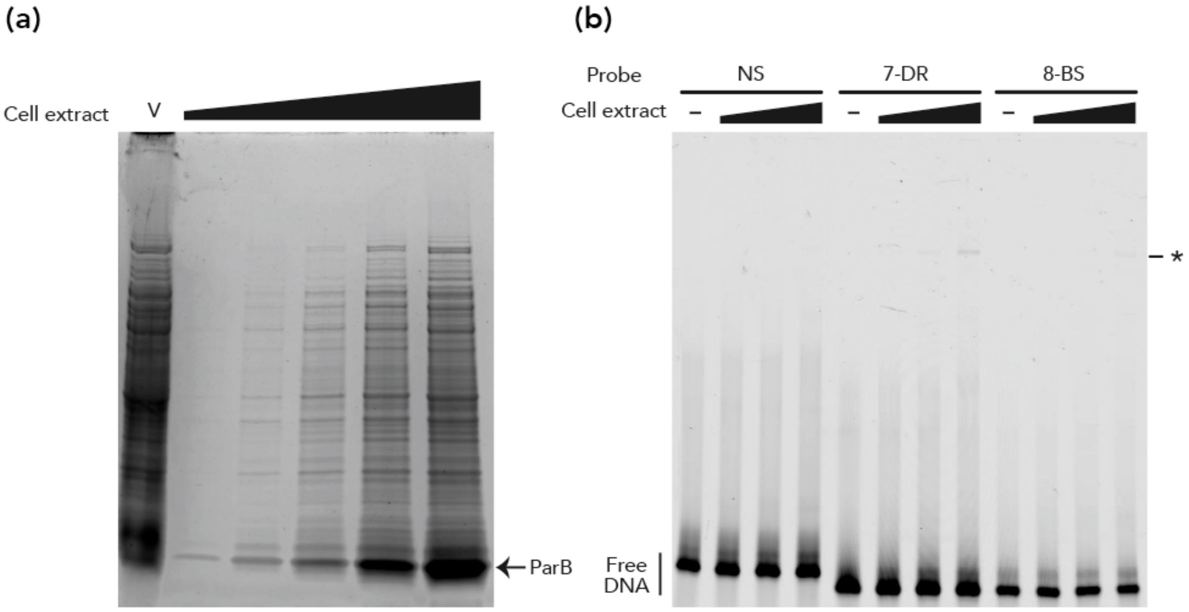

Figure S1. Crude cellular extracts and EMSA in the absence of ParB. (**a**) SDS-PAGE followed by Coomassie blue staining of an increasing range (1, 3, 10, 30 and 100; black triangle) of total extracts from cells expressing $ParB_{pESBL}$. The cell extract from the vector control (V) is loaded at the corresponding range 100. The position of ParB is indicated by the arrow. (**b**) Cy3-labeled dsDNA probes were incubated without (–) or with increasing amounts of cell extract from the vector control (range 10, 30, 100; black triangle). Free DNA fragments of the NS, 7-DR and 8-BS probes were indicated on the left. A faint non-specific complex, labeled with an asterisk (*) is visible at high cell extracts with the 7-DR and 8-BS but not with the NS probes.

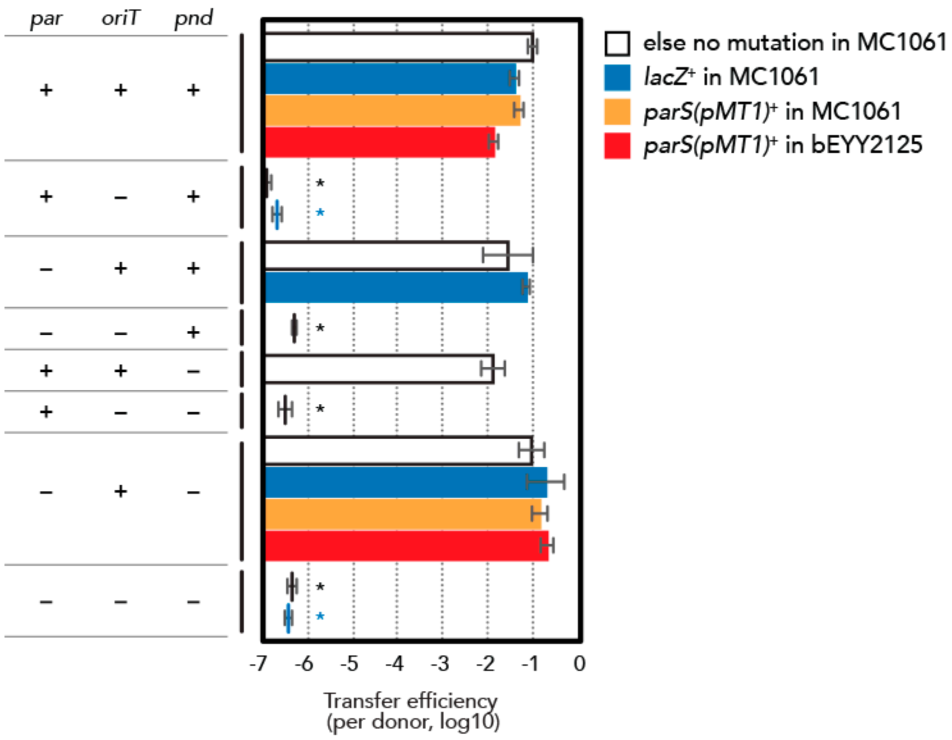

Figure S2. Transfer efficiency of different pESBL mutants. The presence (+) or absence (–) of *par*, *oriT* and *pnd* genes are indicated on the left, and additional characteristics in pESBL and donor *E. coli* are indicated with colored bars. Exconjugants were never obtained in all *oriT* mutants (*), thus the limit of detection was presented with vertical lines. Average and standard deviations for more than three independent experiments are displayed.

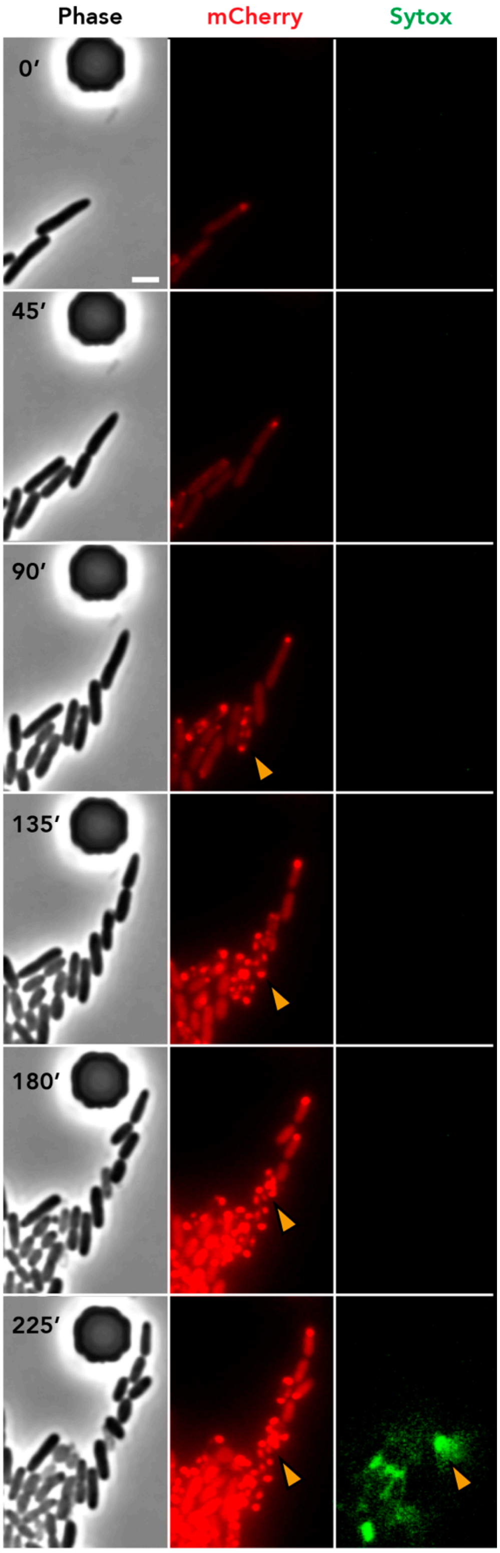

Figure S3. Representative time-lapse images of the fate of cells that have lost Δ*par* Δ*pnd parS*$_{\mathrm{pMT1}}$$^{+}$ pESBL. The cell culture was loaded in a microfluidic chamber, and Amp and Sytox green nucleic acid stains were added in the flow. Arrowheads represent a cell that loses the plasmid, resulting in the aberrant accumulation of mCherry and sensitivity to Amp, which is reflected by the lysis of the cell and release of the chromosome DNA in the media stained by sytox. Bars = 2 μm.

Table S1: Strains used in this study

Strain	Description	Reference
Host E. coli *strains*		
BL21 (DE3)	Host cell for T7 promoter-based protein expression	Laboratory stock
BW25113	F$^-$ DE*(araD-araB)567 lacZ4787(del)::rrnB-3* LAM$^-$ *rph-1* DE*(rhaD-rhaB)568 hsdR514*	[20]
MC1061	*hsdR2 hsdM+ hsdS+ araD139 Δ(ara-leu)7697 Δ(lac)X74 galE15 galK16 rpsL mcrA mcrB1*	Laboratory stock
SM10 λ*pir*	*thi thr leu tonA lacY supE recA::RP4-2-Tc::Mu_kan λpir*	Laboratory stock
β2163	*F*$^-$ *RP4-2-Tc::Mu_kan ΔdapA::(erm-pir)*	[62]
YBA268	MC1061 / pXX705	[63]
bEYY2082	MC1061 / pEYY367	This study
bEYY2092	MC1061 / pEYY378	This study
bEYY2118	BW25113 *ΔgalK::mcherry-parB*$_{pMT1}$*_kan*	This study
bEYY2125	MC1061 *ΔgalK::mcherry-parB*$_{pMT1}$*_kan*	This study
Strains for allelic exchange		
bEYY1101	SM10 λ *pir* / pEYY40	This study
bEYY1204	β2163 / pEYY84	This study
bEYY2009	SM10 λ *pir* / pEYY352	This study
bEYY2060	SM10 λ *pir* / pEYY371	This study
bEYY2091	SM10 λ *pir* / pEYY377	This study
Strains harboring pESBL		
YBB1195	MC1061 / pESBL	[14]
bEYY1116	MC1061 / pESBL Δ*oriT*	This study
bEYY1230	MC1061 / pESBL *hp4::lacZ_spc*	This study
bEYY1438	MC1061 / pESBL *ΔoriT hp4::lacZ_spc*	This study
bEYY2026	MC1061 / pESBL *Δpar*	This study
bEYY2051	MC1061 / pESBL *ΔoriT Δpar*	This study
bEYY2072	MC1061 / pESBL *IG_hp23::parS*$_{pMT1}$	This study
bEYY2093	MC1061 / pESBL *Δpnd*	This study
bEYY2094	MC1061 / pESBL *ΔoriT Δpnd*	This study
bEYY2095	MC1061 / pESBL *Δpar Δpnd*	This study
bEYY2096	MC1061 / pESBL *ΔoriT Δpar Δpnd*	This study
bEYY2115	MC1061 / pESBL *Δpar Δpnd IG_hp23::parS*$_{pMT1}$	This study
bEYY2127	bEYY2125 / pESBL *IG_hp23::parS*$_{pMT1}$	This study
bEYY2166	bEYY2125 / pESBL *Δpar Δpnd IG_hp23::parS*$_{pMT1}$	This study
bEYY2167	bEYY2125 / pESBL *ΔoriT Δpar Δpnd IG_hp23::parS*$_{pMT1}$	This study
bEYY2202	MC1061 / pESBL *Δpar Δpnd hp4::lacZ_spc*	This study
bEYY2203	MC1061 / pESBL *ΔoriT Δpar Δpnd hp4::lacZ_spc*	This study
bEYY2242	MC1061 / pESBL *Δpar hp4::lacZ_spc*	This study

Table S2: Plasmids used in this study

Plasmid	Description	Construction/reference
Vectors		
pBAD33	p15A*ori cat* P_{ara}*_X*	[64]
pDM4	R6K*ori cat sacB* mob_{RP4}	[65]
pKD46	*oriR101 repA101ts bla* P_{ara}*_exo_bet_gam*	[20]
pXX705	F*ori amp-R ΔsopABC*	[29]
pEYY240	pBAD33 *X-m.sf.gfp-mut3*	*m.sf.gfp.mut3* amplified with oYo578 x oYo579 followed by SacI + XbaI digestion cloned into pBAD33 / SacI + XbaI
Allelic exchange plasmids		
pEYY10	pDM4 for *hp4::X*	3 pieces Gibson assembly: left arm amplified with oYo17 x oYo24, right arm amplified with oYo25 x oYo20 and pDM4 / XbaI + XhoI
pEYY40	pDM4 for *ΔoriT*	3 pieces Gibson assembly: left arm amplified with oYo107 x oYo108, right arm amplified with oYo110 x oYo111 and pDM4 / XbaI + XhoI
pEYY84	pDM4 for *hp4::lacZ_spc*	3 pieces Gibson assembly: *spc* from pVI36 amplified with oYo226 x oYo227, *lacZ* amplified with oYo228 x oYo229 and pEYY10 / XbaI
pEYY352	pDM4 for *Δpar*	3 pieces Gibson assembly: left arm amplified with oYo881 x oYo873, right arm amplified with oYo880 x oYo879, and pDM4 / XbaI + XhoI
pEYY371	pDM4 for *IG_hp23::*$parS_{pMT1}$	4 pieces Gibson assembly: $parS_{pMT1}$ amplified with oYo946 x oYo947, left arm amplified with oYo944 x oYo945, right arm amplified with oYo948 x oYo949 and pDM4 / XbaI + XhoI
pEYY377	pDM4 for *Δpnd*	3 pieces Gibson assembly: left arm amplified with oYo1026 x oYo1027, right arm amplified with oYo1028 x oYo1029, and pDM4 / XbaI + XhoI
Other plasmids		
pEE18	R6K*ori* mob_{RP4} *cat magellan5-MmeI (kan)*	pEE22 [22] but without SC2 reporter
pSN70	pUC*ori bla* $lacI^q$ P_{lac}*_mcherry-*$ParB_{pMT1}$[1]	[39]
pR6K biofab-sf.gfp	R6K*ori FRT_kan_FRT* PB_{iofab}*_sf.gfp*	Gibson Assembly of annealed oligo nucleotides ol307 + ol308 and PCR fragment from pR6KplaciQ1-sfGFP [39] with ol305 x ol306
pVI36	R6K*ori bla FRT_spc_FRT*	[63]
pEYY367	pBAD33 *m.sf.gfp-mut3-*$parB_{pESBL}$	Gibson assembly of $parB_{pESBL}$ *(hp7)* amplified with oYo960 x oYo961 and pEYY240 /NotI + XbaI
pEYY373	R6K*ori FRT_kan_FRT* PB_{iofab}*_ mcherry-*$ParB_{pMT1}$[1]	Gibson assembly of vector from pR6K biofab-sf.gfp amplified with oYo998 x oYo999 and *mCherry-parBpMT1* amplified from pSN70 with oYo1000 x oYo1001
pEYY378	pXX705 par_{pESBL}	Gibson assembly of *par* locus amplified with oYo1032 x oYo1033 and pXX705 / BamHI
pEYY395	pET28b $parB_{pESBL}$	Gibson assembly of $parB_{pESBL}$ *(hp7)* amplified with oYo1092 x oYo1094 and pET28b / HindIII + NcoI

[1] ParB $_{pMT1}$ lacks N-terminal region to (pMT1 Δ23ParB, see [33]) to avoid dimerization and interaction to ParA.

Table S3: Oligo DNAs used in this study

Oligo	Sequence (5′ -> 3′)
oYo17	GCGGAGTGTATATCAAGCTTATCGAAGTGATTCGGGAGCTTAGC
oYo20	TTGTGAGCGGATAACAATTTGTGGGAGCCCTTGCTGTGACAATG
oYo24	GACCTTTCAGACATTCCAGGTCTAGAATGGCATCAAGACACTCACG
oYo25	CGTGAGTGTCTTGATGCCATTCTAGACCTGGAATGTCTGAAAGGTC
oYo107	GCGGAGTGTATATCAAGCTTATCGAAAGCCCTGGTATTTATGCC
oYo108	CACTCACTTCAGGCTCCTTACTAGTCTTATGCAGACGGCAG
oYo110	CTGCCGTCTGCATAAGACTAGTAAGGAGCCTGAAGTGAGTG
oYo111	TTGTGAGCGGATAACAATTTGTGGTTGACCGCAACGTGAACATG
oYo226	CGTGAGTGTCTTGATGCCATTTAGCTTGCAGTGGGCTTAC
oYo227	GAGGTCGATATTGACCCAATTCTCATTGGCTGGCACCAAGC
oYo228	GCTTGGTGCCAGCCAATGAGAATTGGGTCAATATCGACCTC
oYo229	GACCTTTCAGACATTCCAGGTTGGCTTATTGTGGGGATGAC
oYo578	AATTCGAGCTCAAGGAGGAAAACCATGAGTAAAGGTGAAGAACTGTTC
oYo579	ACTCTAGATTAGGCGGCCGCCGCTTTGTAGAGTTCATCCATGC
oYo873	TTGTGAGCGGATAACAATTTGTGGTGATGAAATCCAGCCCCG
oYo879	GCGGAGTGTATATCAAGCTTATCGCCGGCTGGCTGGTTTATT
oYo880	CGACGCAGATGACCAGAAAACAGAGGTATCACGATTGATAGCA
oYo881	TGCTATCAATCGTGATACCTCTGTTTTCTGGTCATCTGCGTCG
oYo944	GCGGATAACAATTTGTGGCGACACCTGTCCTGAA
oYo945	ATTTCGCGCTCTGTTTAATCAGACTCGTGGC
oYo946	TTAAACAGAGCGCGAAATTATGAGTCACG
oYo947	CAGGGCTTTAGGATGCCGAAGAGC
oYo948	CGGCATCCTAAAGCCCTGGTATTTATGCC
oYo949	GGAGTGTATATCAAGCTTATCGTAGTCTTATGCAGACGGC
oYo960	TCTACAAAGCGGCGGCCGCCAGAAATAGAGCAAACACTTCACC
oYo961	TGCCTGCAGGTCGACTCTAGATTATCTTTGTTTTACTTCTGCTATCAA
oYo998	GGTGAGTAACCCGGGTGTAGGCTG
oYo999	GCTCACCATATTCACCACCCTGAATTGAC
oYo1000	GTGGTGAATATGGTGAGCAAGGGCG
oYo1001	TACACCCGGGTTACTCACCTGATTCTGGAAG
oYo1026	TTGTGAGCGGATAACAATTTGTGGCATCACTGATAATGTCCTCGC
oYo1027	GTCAGCCTTCGCAACAAAGCCCGCAGCTATTCTAACG
oYo1028	CGTTAGAATAGCTGCGGGCTTTGTTGCGAAGGCTGAC
oYo1029	GCGGAGTGTATATCAAGCTTATCGCCCACTCTGTAACGGAAC
oYo1032	GACATCCAGCCTGCTGTTGGGTTCCACTGAGCGTCAGACCC
oYo1033	CTCGTTTCTGACACTTGCAGACTGACAGATTTGCCAGTAGCC
oYo1092	GGGCCATGGTGAGAAATAGAGCAAACACTTCACC
oYo1094	CCCAAGCTTTTATCTTTGTTTTACTTCTGCTATCAA
ns-F	Cy3-CCGTTCTAGCTCATTCTGTTCTTGCTTGCTGCATCATCACTGCATCATCCCAATGCCG ATCTAGCTCATTACTGTTCTAT
ns-R	ATAGAACAGTAATGAGCTAGATCGGCATTGGGATGATGCAGTGATGATGCAGCAAGCA AGAACAGAATGAGCTAGAACGG
7DR-F	Cy3-CCATAATATTGTTATCTTGTTATCTTGTTATCTTGTTATCTTGTTATCTTGTTGTCTTGT TATCTTAATTAA
7DR-R	TTAATTAAGATAACAAGACAACAAGATAACAAGATAACAAGATAACAAGATAACAAG ATAACAATATTATGG
8BS-F	Cy3-TTGAGATAACAAGATAACAAGATAACAAGATAACACCTTGTTATCTTGTTATCTTG TTATCTTGTCATCGAC
8BS-R	GTCGATGACAAGATAACAAGATAACAAGATAACAAGGTGTTATCTTGTTATCTTGTTAT CTTGTTATCTCAA
ol305	TATAATAGATTCATGGATGCAAAATAAGAGAGTCAATTCAGGGTGGT
ol306	GCGAAGTCAATACTCTATCGATGCTTCCAGTCGGGAAACCTGTCGTG
ol307	GCATCGATAGAGTATTGACTTCGCATCTTTTTGTACCTATAATAGATTCATGGATGCAAA AT
ol308	ATTTTGCATCCATGAATCTATTATAGGTACAAAAAGATGCGAAGTCAATACTCTATCGA TGC

Table S4: Summary of Tnseq

Experiment		Illumina barcode[1]	P5 adaptor[2]	# of reads				
				Raw reads	Bowtie-mapped	TA-mapped		
						Total	Chr[3]	pESBL
Input library	#1	41	ATGCTA	15,444,882	16,905,660	14,040,424	13,956,375	84,049
	#2	1	CAGT	14,776,122	15,414,730	15,214,960	15,188,012	26,948
	#3	8	ACAGT	12,687,763	12,876,419	12,699,636	12,654,016	45,620
	Total					41,955,020	41,798,403	156,617
Output library	#1	42	AGCATA	14,389,584	14,691,470	14,516,201	14,480,809	35,392
	#2	5	CGTA	18,531,553	20,124,007	19,864,167	19,765,407	98,760
	#3	9	TACTC	14,609,016	15,189,471	14,970,843	14,902,612	68,231
	Total					49,351,211	49,148,828	202,383

[1] Index number (visit https://support.illumina.com for details). [2] See [22]. [3] Chromosome.
